# Investigating the change in gene expression profile of blood mononuclear cells post-laparoscopic sleeve gastrectomy in Chinese obese patients

**DOI:** 10.3389/fendo.2023.1049484

**Published:** 2023-03-14

**Authors:** Na Liu, Xiaolei Chen, Jianghua Ran, Jianhui Yin, Lijun Zhang, Yuelin Yang, Jianchang Cen, Hongmei Dai, Jiali Zhou, Kui Gao, Jihong Zhang, Liyin Liu, Zhiyuan Chen, Haibin Wang

**Affiliations:** ^1^ Department of Endocrinology, The First People’s Hospital of Kunming, Kunming, Yunnan, China; ^2^ Department of General Surgery, The First People’s Hospital of Kunming, Kunming, Yunnan, China; ^3^ Clinical Laboratory, The First People’s Hospital of Kunming, Kunming, Yunnan, China; ^4^ Department of Vascular Surgery, The First People’s Hospital of Kunming, Kunming, Yunnan, China; ^5^ Ophthalmology, The First People’s Hospital of Kunming, Kunming, Yunnan, China; ^6^ Faculty of Physical Education, Kunming University of Science and Technology, Kunming, Yunnan, China; ^7^ Department of Infectious Diseases, The Affiliated Hospital of YunNan University, Kunming, Yunnan, China

**Keywords:** laparoscopic sleeve gastrectomy (LSG), obesity, bioinformatics, high-throughput sequencing, leptin

## Abstract

**Background:**

Laparoscopic sleeve gastrectomy (LSG) is a sustainable technique that effectively treats morbid obesity. However, the molecular mechanisms underlying the improvement of metabolic health following this process warrants more investigation. This study investigates LSG-related molecules and uses bulk RNA-sequencing high-throughput analysis to unravel their regulatory mechanisms.

**Methods:**

Peripheral blood mononuclear cells (PBMC) were collected from ten obese patients with BMI ≥ 32.5 kg/m^2^ in the Department of General Surgery of Kunming First People’s Hospital. After LSG, patients were followed up for one month, and blood samples were retaken. Blood samples from ten patients before and after LSG and bulk RNA-Seq data were analyzed in this study. LSG-associated gene expression was detected by weighted gene coexpression network analysis (WGCNA) and differential analysis. Subsequently, essential signature genes were identified using logistic least absolute shrinkage and selection operator (LASSO) and support vector machine-recursive feature elimination (SVM-RFE) algorithms. Gene Ontology (GO), Kyoto Encyclopedia of Genes and Genomes (KEGG), and single-sample gene set enrichment analysis (ssGSEA) were utilized to reveal the potential functions of the target genes. Furthermore, the Pearson correlation of signature genes with leptin and lipocalin was also explored. Finally, we constructed a robust endogenous RNA (ceRNA) network based on miRWalk and starBase databases.

**Results:**

We identified 18 overlapping genes from 91 hub genes, and 165 differentially expressed mRNAs (DE-mRNA), which were revealed to be significantly associated with immune cells, immune response, inflammatory response, lipid storage, and localization upon functional enrichment analysis. Three signature genes, *IRF1*, *NFKBIA*, and *YRDC*, were identified from the 18 overlapping genes by LASSO and SVM-REF algorithms. The logistic regression model based on the three signature genes highlighted how robustly they discriminated between samples. ssGSEA indicated these genes to be involved in lipid metabolism and degradation pathways. Moreover, leptin levels were significantly reduced in patients undergoing LSG, and *NFKBIA* significantly negatively correlated with leptin. Finally, we identified how the long non-coding RNA (lncRNA) *ATP2B1-AS1* regulated the expression of the signature genes by competitively binding to six microRNAs (miRNAs), which were hsa-miR-6509-5p, hsa-miR-330-5P, hsa-miR-154-5P, hsa-miR-145-5P, hsa-miR4726-5P and hsa-miR-134-5P.

**Conclusion:**

This study identified three critical regulatory genes significantly differentiated between patients before and after LSG treatment and highlighted their potentially crucial role after bariatric surgery. This provides novel insights to increase our understanding of the underlying mechanisms of weight loss and associated metabolic improvement after bariatric surgery.

## 1 Introduction

Morbid obesity is a chronic disease threatening human health and life all over the world. Nowadays, obesity is increasing rapidly, leading to an outbreak trend (1, 2). Over the past few decades, the overweight and obese population has grown significantly in most Asian countries (3). The World Health Organization (WHO) report of 2016 stated that 650 million adults and more than 340 million children and adolescents were overweight or obese (4). Following the current trend will lead to half of the world’s adult population becoming overweight or obese by 2030 (5). Obesity has a global impact on public health and the economy, posing a burden. According to recent estimates, the suggested cost of treating obesity-related comorbidities has reached a staggering $2 trillion, equivalent to 2.8 percent of the global GROSS domestic product (6).

The first-line treatment for obesity includes managing body weight. Maintaining long-term body weight loss is challenging, especially for severely obese patients who still struggle with the process despite lifestyle adjustments and drug therapy. Bariatric surgery might be the most sustainable and effective quality management strategy for treating severe obesity and its related metabolic diseases (7). However, weight loss mechanisms and the subsequent metabolic improvement after bariatric surgery remain unclear. An in-depth study to identify the underlying mechanisms might be instrumental to fully understanding the pathogenesis of obesity and deciding on weight-loss operations. Bariatric surgery was initially thought to merely reduce energy intake. Progressive evidence shows that the cause of metabolic improvement after weight loss is a culmination of mutual influence and common changes. This process potentially changes the body’s absorption and intake, affecting the gastrointestinal hormone levels, gastrointestinal flora, and adipokines and regulating the central feeding system. Studies have shown that bariatric surgery reduces patients’ weight and improves the treatment of diabetes and cardiovascular disease, and even reduces cancer incidences and related risks (8, 9). However, the mechanism underlying weight loss and metabolic improvement after weight-loss surgery is still unclear (10).

Several studies also reported inflammatory changes in adipose tissue and blood, particularly at early stages during the first year after surgery. RNA sequencing (RNA-seq) technology is a powerful and reliable tool for profiling gene expression (11), which plays an instrumental role in identifying several biological pathways affected by weight loss. This study analyzed expression profiles from RNA-seq data in peripheral blood from 10 obese patients undergoing bariatric surgery, whose gene expression levels were assessed before and one month after surgery. This approach provided insights into significant changes in transcriptome profiles and underlying molecular mechanisms disrupting biological pathways. Our study indicated that improving the inflammatory state after bariatric surgery might be caused by a break in the coexpression between inflammatory signaling pathways and a few crucial molecules involved in chemotaxis and activation of immune cells. So, this study potentially provides novel insights and identifies indicators of weight loss and metabolic improvement after bariatric surgery. The results would significantly contribute to understanding the etiology of obesity, diabetes, and other diseases and search for potential biomarkers for better therapy.

## 2 Materials and methods

### 2.1 Patient recruitment and clinical characteristics

According to the Chinese Guidelines for Surgical Treatment of Obesity and Type 2 Diabetes (2019 Edition), inclusion criteria for this study included indications for bariatric surgery, BMI ≥32.5 kg/m^2^. The exclusion criteria included a history of previous bariatric surgery, gastrectomy, substance abuse, uncontrolled mental illness, end-stage organ disease, or advanced cancer. Ten obese patients who received bariatric surgery in The Department of General Surgery of Kunming First People’s Hospital were randomly selected from January 2020 to January 2021. The clinical information is shown in **Table 1**. The age of the patients was 35 ± 15 years, with or without abnormal glucose metabolism. There was no significant difference in drug application status since they were not taking any that affected inflammatory pathways, glucose homeostasis, or fatty acid metabolism. Follow-up was performed one month after surgery. The Ethics Committee of the hospital approved this study. The ethical clearance numbe is YLS2020-45. All patients signed an informed consent form.

**Table 1:** Characteristics of the studied patients.

Gender	Age	Weight(kg)		BMI(kg/m^2^)		FPG(mmol/L)		Cortisol(nmol/L)		ACTH(pmol/L)	
		pre-LSG	post-LSG	pre-LSG	post-LSG	pre-LSG	post-LSG	pre-LSG	post-LSG	pre-LSG	post-LSG
F	37	109	93	44.22	37.73	5.1	5.31	227.1	171.4	1.31	1.77
M	20	120	99	44.08	36.36	4.96	5.00	335	173.9	7.44	5.18
M	38	101	88	33.75	29.40	5.84	5.95	453	320.5	5.53	4.96
F	27	90.2	82	33.95	30.86	5.6	4.79	103.1	273.9	1.1	1.33
F	34	95	86	35.76	32.37	5.7	5.16	237.2	160.6	3.22	1.33
F	34	135	113	51.44	43.06	5.23	5.6	168	475.2	2.11	4.86
F	49	102	89	34.48	30.08	8	7.26	273.4	234.2	3.85	2.3
F	39	104	89.2	38.67	33.16	6.2	6.46	258	228.1	3.18	3.02
F	29	115	102	41.73	37.02	4.34	4.5	312	283.4	5.25	5.2
F	36	121	92	46.11	35.06	4.61	5	319	211	6.43	4.07

(F, female; M, male; LSG, Laparoscopic Sleeve Gastrectomy).

### 2.2 High-throughput bulk RNA-seq

Total RNA was isolated and purified using TRIzol reagent (Invitrogen, Carlsbad, CA, USA) following the manufacturer’s procedure. The amount and purity of each RNA sample were quantified using NanoDrop ND-1000 (NanoDrop, Wilmington, DE, USA). The RNA integrity was assessed by Bioanalyzer 2100 (Agilent, CA, USA) (RIN values greater than 7.0 met the requirements RIN number >7.0) and confirmed by electrophoresis with denaturing agarose gel. Poly (A) RNA was purified from 1 μg total RNA using Dynabeads Oligo (dT)25-61005 (Thermo Fisher, CA, USA) using two rounds of purification.

The poly (A) RNA was fragmented into small pieces using Magnesium RNA Fragmentation Module (NEB, cat.e6150, USA) for 94°C 5-7min. Then, the cleaved RNA fragments were reverse-transcribed to create the cDNA by SuperScript™ II Reverse Transcriptase (Invitrogen, cat. 1896649, USA), which were then used to synthesize U-labeled second-stranded DNAs with E. coli DNA polymerase I (NEB, cat.m0209, USA), RNase H (NEB, cat.m0297, USA) and dUTP solution (Thermo Fisher, cat.R0133, USA). An A-base was then added to the blunt ends of each strand, preparing them for ligating to the indexed adapters. Each adapter contained a T-base overhang for ligating the adapter to the A-tailed fragmented DNA. Single- or dual-index adapters are ligated to the fragments, and size selection was performed with AMPureXP beads. After the heat-labile UDG enzyme (NEB, cat.m0280, USA) treatment of the U-labeled second-stranded DNAs, the ligated products were amplified with PCR by the following conditions: initial denaturation at 95°C for 3 min; 8 cycles of denaturation at 98°C for 15 sec, annealing at 60°C for 15 sec, and extension at 72°C for 30 sec; and then final extension at 72°C for 5 min. The average insert size for the last cDNA library was 300 ± 50 bp. Finally, we performed the 2×150 bp paired-end sequencing (PE150) on an Illumina Novaseq™ 6000 (LC-Bio Technology CO., Ltd., Hangzhou, China) following the vendor’s recommended protocol.

### 2.3 Weighted gene coexpression network analysis

We used the R package WGCNA (Version 1.70-3) (11) to detect gene coexpression modules associated with a cohort of obese patients before and after receiving laparoscopic sleeve gastrectomy (LSG) treatment. Sample clustering helped to detect outliers and to match samples to their features. Module-trait correlation analysis was applied to detect coexpression modules with the highest correlation to clinical features. Correlation analysis of gene significance (GS) and module membership (MM) helped identify the hub genes; | MM| > 0.8 and |GS| > 0.2 were the thresholds. The higher the correlation between GS and MM, the greater the importance of the gene for the trait and the module (12).

### 2.4 Differential analysis

Differential expression analysis was performed to identify the differentially expressed mRNAs (DE-mRNAs) between preoperative and postoperative samples using the R package limma (Version 3.46.0) (13). The selection criteria for significantly differentially expressed mRNAs were |log2 fold change (FC)| > 0.5 and *P* < 0.05.

### 2.5 Screening for signature genes

The common genes were identified *via* overlapping hub genes and DE-mRNAs. Subsequently, the least absolute shrinkage and selection operator (LASSO) and support vector machine-recursive feature elimination (SVM-REF) algorithms were conducted on these common genes, respectively. LASSO Cox regression using the R package glmnet (Version 4.0-2), with penalty parameters, was estimated by 10-fold cross-validation (14). The SVM-REF (15) algorithm analysis was performed based on the R package el070 (Version 1.7-4). The LASSO and SVM-REF models were assessed using receiver operating characteristic (ROC) curves. The signature genes were screened by comparing and selecting the common output of the LASSO model and SVM-REF algorithms. ROC curves were plotted to evaluate diagnostic values of signature genes.

### 2.6 Single-sample gene set enrichment analysis

Single-sample GSEA was performed on signature genes. The ‘c2.cp. Kegg.v7.2.symbols.gmt’ were downloaded from the Molecular Signature Database (MSigDB, http://www.gsea-msigdb.org/gsea/msigdb/) and used as the predefined gene set. We calculated the correlations of the signature genes with all other genes separately and ranked their correlations from highest to lowest. The KEGG (Kyoto Encyclopedia of Genes and Genomes) gene sets were deployed as a test set to detect the enrichment of signaling pathway in them. Pathways that met all three conditions simultaneously were identified as significant, |normalized enrichment score (NES)| ≥ 1, NOM p-val < 0.05, and FDR q-val < 0.25.

### 2.7 Construction of competitive endogenous RNA network

We constructed a regulatory network of DE-lncRNAs-miRNAs-signature genes based on the identified signature genes. The reciprocal microRNAs (miRNAs) of the signature genes were predicted by the miRWalk database (http://mirwalk.umm.uni-heidelberg.de/). Subsequently, lncRNAs interacting with the above miRNAs were retrieved from the starBase2.0 database (http://starbase.sysu.edu.cn/index.php). Then, the DE-lncRNAs were selected from the retrieved lncRNAs that were consistent with the expression trends of the signature genes to be consistent with the ceRNA mechanism. The ceRNA network was visualized by the R package Cytoscape.

### 2.8 Detection of leptin and adiponectin levels

Around 5 ml venous blood was collected from patients after fasting and centrifuged at 1000 g for 10 min. The plasma was separated and kept at -80°C. A double antibody sandwich ELISA(Wuhan MSK Biotechnology Co. LTD) was used to detect the plasma levels of leptin and Adiponectin.

### 2.9 Statistical analysis

Correlation coefficients of the signature genes with leptin and lipocalin were calculated by Pearson correlation analysis, where the Wilcoxon rank-s test compared the changes in leptin and lipocalin in patients before and after LSG. Ingenuity Pathway Analysis (IPA) revealed the diseases and functions involved in DE-mRNAs and demonstrated the interaction network of signature genes. Overlap analysis was performed using the online tool Jvenn (http://jvenn.toulouse.inra.fr/app/example.html). All analyses and statistics were performed in the R software. If not specified above, p < 0.05 was regarded as statistically significant.

## 3 Results

### 3.1 Identification of hub genes related to obese patients treated with LSG

**Figure 1:**
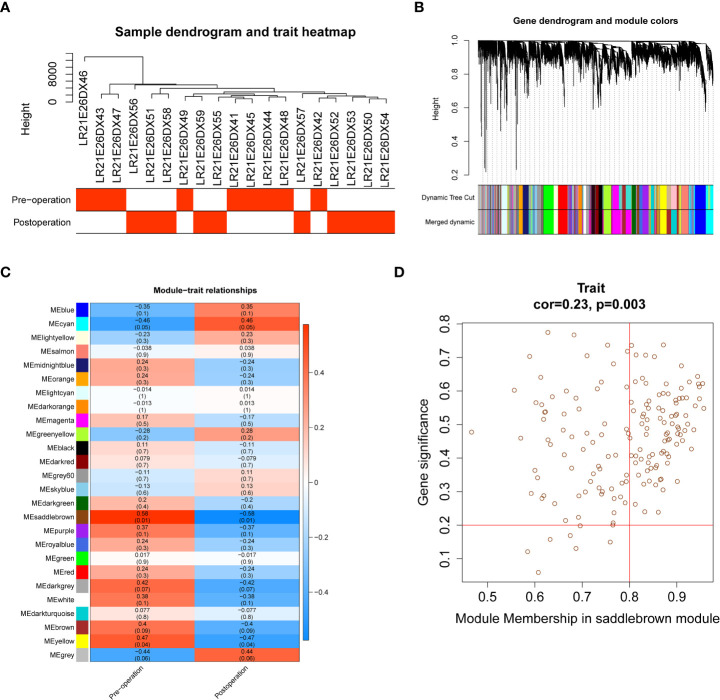
Identification of 91 hub genes related to obese patients treated with LSG by weighted gene coexpression network analysis (WGCNA). **(A)** Sample clustering with preoperative and postoperative external traits. **(B)** Dendrogram of the WGCNA modules. **(C)** The relationship between coexpression modules and external clinical traits and the saddle brown module was considered most relevant to clinical characteristics (cor = 0.58, p = 0.01). **(D)** Scatter plot of module eigengenes in the saddle brown module, the genes with |MM| > 0.8 and |GS| > 0.2 were selected as hub genes (cor = 0.23, p = 0.0.003). The horizontal axis represents the absolute value of the correlation between genes and modules, and the vertical axis represents the absolute value of the correlation between genes and traits.

We performed WGCNA using second-generation sequencing data from ten obese patients before and after LSG, after removing one obvious outlier (No. LR21E26DX40, obese sample before receiving LSG treatment; **Supplementary Figure 1A**). A soft threshold = 14 was chosen to construct a scale-free network (**Supplementary Figures 1B, C**). Similar modules, segmented by the dynamic tree-cutting algorithm, were subsequently merged according to MEDissThres=0.15 (**Supplementary Figures 1D, E**), resulting in 26 modules (**Figures 1A, B**). Our intention to annotate the phenotypes of the modules led us to jointly analyze the two features (pre- and postoperative) and all the genes with the modules in the heatmap (**Figure 1C**). The brown saddle module had the highest correlation with postoperative obese patients (cor = -0.58, *P* = 0.01). At the same time, this module was the most highly correlated with preoperative (cor = 0.58, *P* = 0.01) (**Figure 1C**). Subsequently, 91 out of 163 genes within this module with correlation cutoff values of |MM| > 0.8 and |GS| > 0.2 were selected as hub genes based on the analysis of MM of saddle brown modules for GS (**Figure 1D**; **Supplementary Table 1**).

### 3.2 Identification of DE-mRNAs after LSG in obese patients

**Figure 2:**
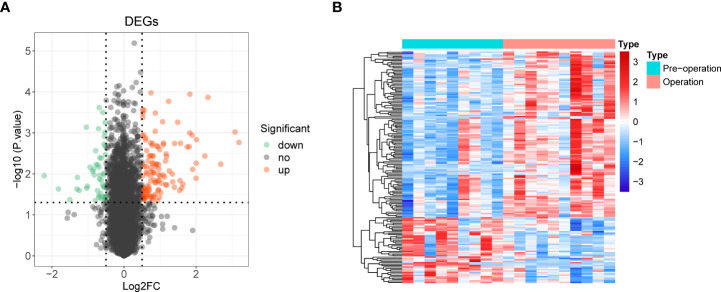
Identification of 165 differentially expressed mRNAs (DE-mRNA) and functional enrichment analysis. **(A)** Volcano plot of DE-mRNAs with |log2 fold change (FC)| > 0.5 and p < 0.05. Red represented upregulated genes, and green indicated down-regulated genes. **(B)** Heat map of DE-mRNAs.

We used the R package limma to identify 165 DE-mRNAs between nine preoperative and ten postoperative samples (**Supplementary Table 2**). Among them, 118 mRNAs were upregulated, and 47 were down-regulated (**Figures 2A, B**). Further, we performed a functional enrichment analysis to reveal the potential functions of these DE-mRNAs (**Supplementary Table 3**).

### 3.3 Identification and evaluation of key biological indicators in obese patients

**Figure 3:**
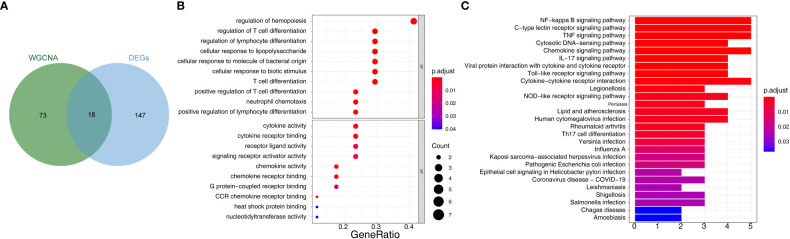
Identification of 18 common 28 bariatric surgery-related genes and functional enrichment analysis of these genes. **(A)** The Venn diagram of 18 common genes 28 key biological indicators between 91 hub genes and 165 DE-mRNAs. **(B)** Gene Ontology (GO) enrichment analysis of bariatric surgery-related genes. **(C)** Kyoto Encyclopedia of Genes and Genomes pathway **(KEGG)** enrichment analysis of bariatric surgery-related genes.

Overlap analysis yielded eighteen common genes from the hub genes and DE-mRNAs (**Figure 3A**; **Supplementary Table 4**). Moreover, GO-BP revealed the eighteen common genes linked to migration, differentiation, and regulation of chemotaxis of immune cells. They were also involved in the inflammatory response, immune response, lipid storage, and localization. Moreover, these genes played essential roles in the molecular functions of chemokine and cytokine activity and receptor binding (**Figure 3B**; **Supplementary Table 5**). KEGG analysis indicated that the ‘NF-kappa B signaling pathway’, ‘C-type lectin receptor signaling pathway’, and ‘TNF signaling pathway’ were the three most relevant pathways (**Figure 3C**; **Supplementary Table 6**).

**Figure 4:**
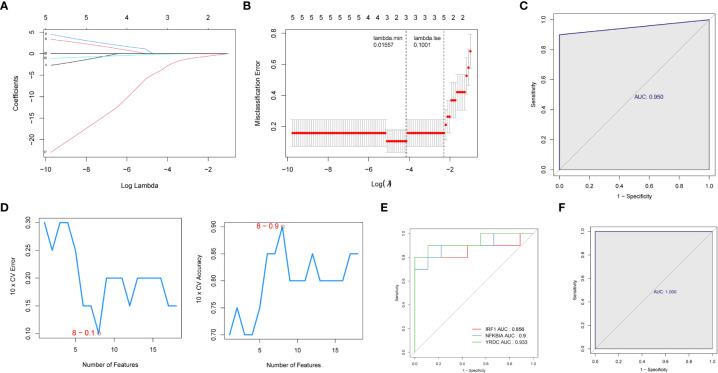
Identification of three signature genes. **(A)** LASSO Cox regression analysis to shrink estimated coefficients to zero. **(B)** Cross-validation was used to choose an optimal λ value in the LASSO selection diagram. The two dotted lines indicated two particular values of λ. The left side was λmin, and the right side was λ1se. The λmin was selected to build the model for accuracy in our study. **(C)** ROC analysis was adopted to assess the efficacy of the LASSO regression analysis results model (AUC = 0.950). **(D)** Plots of eight feature genes selection by SVM-REF. **(E)** ROC curve of three signature genes. **(F)** The ROC curve for L is the logistic regression model of three signature genes (AUC = 1).

We then used two machine learning algorithms, LASSO and SVM-REF, respectively, to further narrow down the biomarkers. Eighteen common genes were subjected to LASSO Cox regression analysis to calculate the regression coefficients. The coefficients for each gene in obese patients are shown in **Figures 4A, B**. The LASSO analysis identified three signature genes *(IRF1*, *NFKBIA*, and *YRDC*) and incorporated them into the classifier, the efficacy of which was evaluated by ROC analysis. The AUC showed that the LASSO algorithm constructed a classifier with an accuracy of up to 0.950 (**Figure 4C**). Moreover, from the eighteen common genes, SVM-REF identified eight as representative feature genes with a minimum generalization error of 0.1 and a maximum accuracy of 0.9 (**Figure 4D**). Ten-fold cross-validation revealed these eight genes to be *IRF1*, *YRDC*, *NLRP3*, *EGR3*, *FCAR_2*, *CCL4L1*, *ZFP36*, and *NFKBIA*. Subsequently, we crossed the feature genes screened by both algorithms and obtained *IRF1*, *NFKBIA*, and *YRDC* as the final feature genes. The performance evaluation analysis of individual feature genes showed their capability to effectively distinguish between pre-LSG and post-LSG obese samples (**Figure 4E**). Moreover, the logistic regression model constructed based on the three final signature genes exhibited robust discriminatory validity (**Figure 4F**). Furthermore, we also predicted the interplay network of three biomarkers by IPA (**Supplementary Figure 2**).

### 3.4 Analysis of potential pathways regulated by the three biomarkers

**Figure 5:**
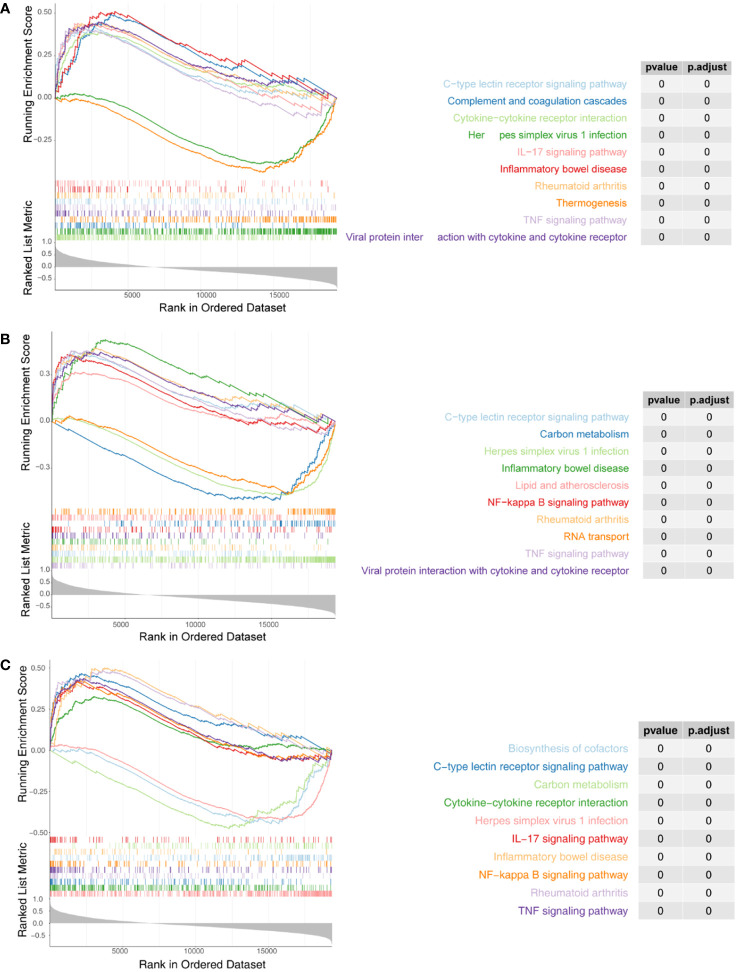
Single-sample Gene set enrichment analysis (ssGSEA) of three biomarkers. **(A)** Enrichment analysis of IRF1 single gene GSEA. **(B)** Enrichment analysis of NFKBIA ssGSEA. **(C)** Enrichment analysis of ssGSEA in YRDC. The screening criteria were set as |normalized enrichment score (NES)| ≥ 1, NOM p-val < 0.05, FDR q-val < 0.25.

We next performed ssGSEA in the R package GSVA to explore the relevant pathways regulated by the three biomarkers. For *IRF1*, a total of 94 KEGG pathways were enriched, of which 57 were enriched in genes positively associated with *IRF1* (NES > 1) and 37 were enriched in genes negatively associated with it (NES < -1) (**Figure 5A**; **Supplementary Table 7**); *NFKBIA* was enriched in a total of 95 KEGG pathways, of which 55 were enriched in genes positively associated with *NFKBIA* and 40 in genes negatively associated with it (**Figure 5B**; **Supplementary Table 8**); A total of 68 KEGG pathways were enriched in *YEDC*, with 50 pathways enriched for genes positively associated with *YEDC* and eighteen for genes negatively associated it (**Figure 5C**; **Supplementary Table 9**).

Comprehensive analysis showed all three biomarkers involved in viral infection-associated diseases such as ‘Herpes simplex virus 1 infection’, ‘Kaposi sarcoma-associated herpesvirus infection’, ‘Human cytomegalovirus infection’, ‘Influenza A’, and ‘Hepatitis C’. Besides, they were associated with immune and inflammatory responses (‘TNF signaling pathway’, ‘IL-17 signaling pathway’, ‘Th17 cell differentiation’, ‘Allograft rejection’, ‘Th1 and Th2 cell differentiation’, etc.). Three biomarkers were significantly enriched in fatty acid metabolism and degradation pathways. Therefore, we hypothesized that the three biomarkers might be involved in the successful weight loss outcome of LSG-treated obese patients by regulating fatty acid metabolism and degradation processes.

**Figure 6:**
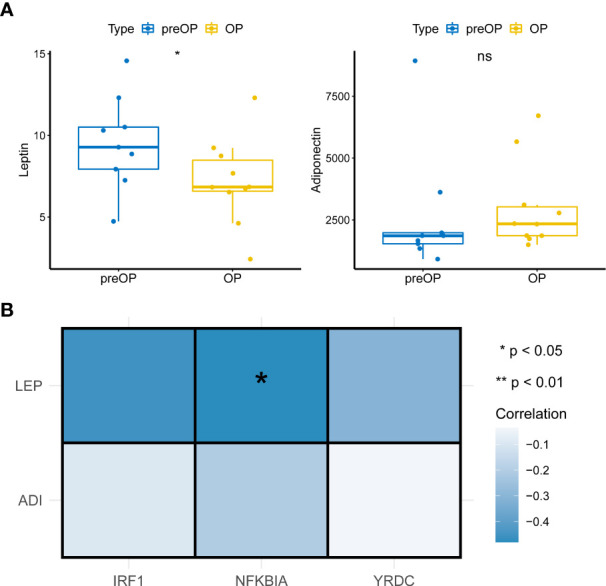
Pearson correlations analysis. **(A)** Box plot of Leptin and Adiponectin expression in patients before and after LSG treatment pre- and post-operation (Wilcoxon rank-sum test). *, *p* < 0.05. **(B)** Pearson correlation analysis of key characteristic genes with leptin and Adiponectin (cor = -0.48, P = 0.038).

The above results led us to explore the changes in leptin and lipocalin in patients before and after LSG. The results showed that LSG significantly decreased leptin levels in patients upon receiving LSG treatment (**Figure 6A**), corroborating a previous study (16). Next, Pearson correlation analysis indicated a significant negative correlation between *NFKBIA* and leptin (cor = -0.48, *P* = 0.038; **Figure 6**), suggesting that the up-regulation of NFKBIA might decrease leptin levels in patients treated with LSG.

### 3.5 A preliminary investigation of biomarker-based ceRNA mechanisms

**Figure 7:**
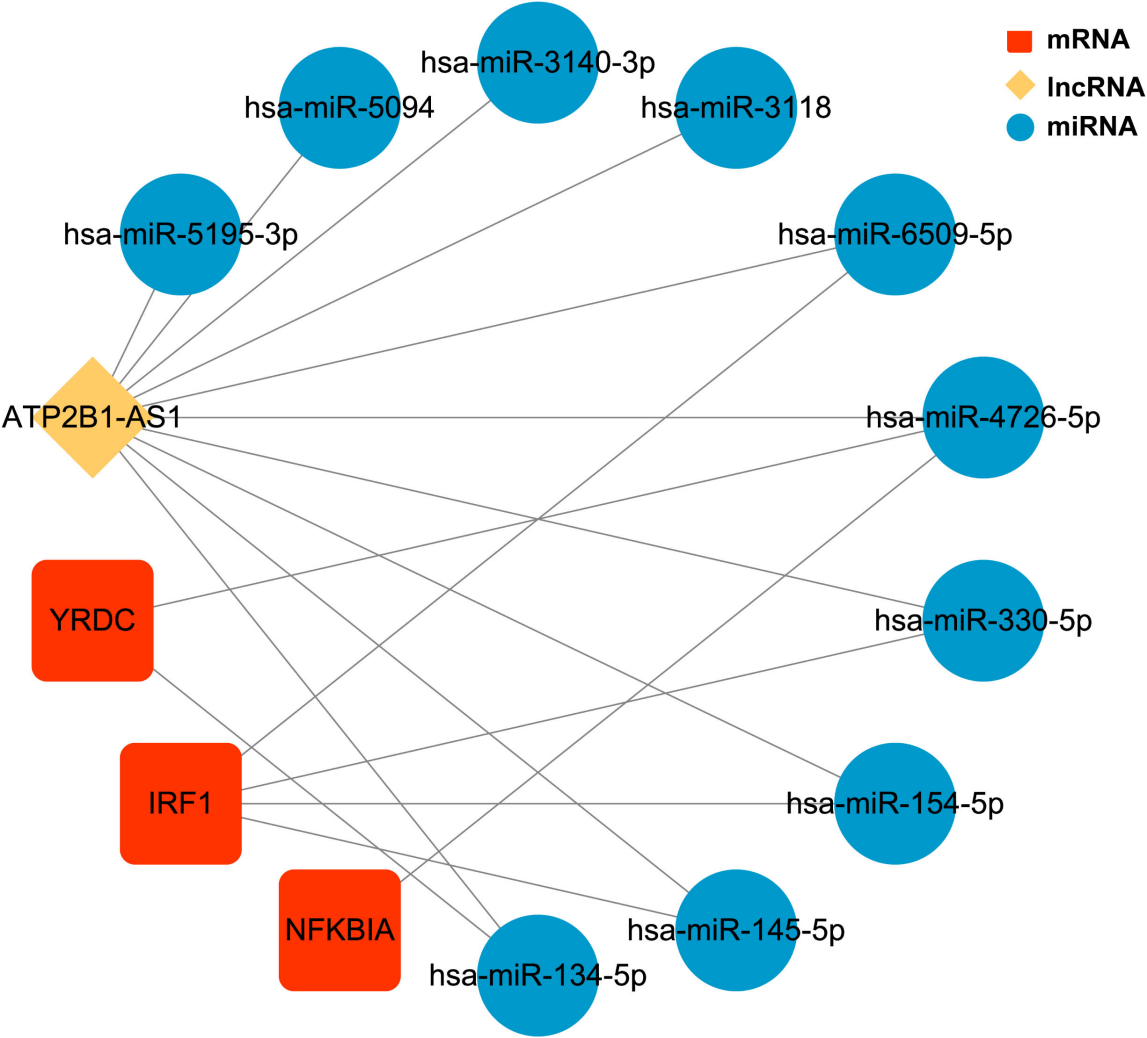
The ceRNA regulatory network of three biomarkers CeRNA network. Red rectangles represent three biomarkers, the yellow diamond represents the lncRNA, and blue circles represent the miRNAs.

We identified 20 upregulated, and two downregulated lncRNAs from obese samples before and after receiving LSG treatment (**Supplementary Table 9**). A ceRNA network based on three biomarkers was subsequently constructed using miRWalk and starBase databases (**Figure 7**; **Supplementary Table 10**). The network included 14 nodes (1 lncRNA, ten miRNAs, and three biomarkers) and 17 edges. The network revealed the regulation of *IRF1* by lncRNA *ATP2B1-AS1* through the competitive binding of 4 miRNAs (*hsa-miR-6509-5P*, *hsa-miR-330-5p*, *hsa-miR-154-5p*, and *hsa-miR145-5P*). lncRNA *ATP2B1-AS1* modulated *NFKBIA* expression through the competitive binding to *hsa-miR4726-5P*, since *YRDC* regulation was achieved by competitive binding of lncRNA *ATP2B1-AS1* to *hsa-miR4726-5P* and/or *hsa-miR-134-5p*.

## 4 Discussion

Morbid obesity is a severe threat to human health, causing debilitating outcomes. Recently, bariatric surgery has emerged as the most effective treatment for morbid obesity (8). Laparoscopic sleeve gastrectomy (LSG) is now the most-performed bariatric procedure worldwide within academic centers (17). However, the critical variables associated with weight loss and metabolic improvement after bariatric surgery remain unclear.

This study detected the differential expressions of the critical biomarkers, IRF1, NFKBIA, and YRDC genes in obese patients after LSG. The enriched pathways associated with these genes included the metabolism and degradation of fatty acids. Our results suggested IRF1, NFKBIA, and YRDC could potentially help in successful weight loss in obese patients treated with LSG. Interferon regulatory factor (IRF) is a transcription factor that regulates the expression of interferon genes. The IRF1 expression has been implicated in the adipocyte inflammatory processes (18). The YRDC is widely expressed in humans, but its biological function is unknown in obese patients. NFKBIA is an inhibitor regulating the movement of the transcription factor NF-κB into and out of the nucleus. It also improves glucose and lipid metabolism in postoperative obese patients which has regulatory effect on diabetes (19, 20). Moreover, the NFKBIA gene might be closely related to postoperative weight loss and improved glucose and lipid metabolism in obese patients.

Our enrichment analyses revealed that the immune cells were closely linked to three characteristic genes and eighteen DE-mRNAs common genes. Changes in the immune micro-environment might play an essential role in obese patients post-LSG. Whole blood enrichment analysis showed that the gene expression had also changed early at one-month post-LSG. Transcriptomic changes occur in inflammatory cytokines and the metabolic pathways associated with energy levels (21). Obesity regulates immune cells in adipose tissue (22). Adipose tissue is a vital immune organ that stores fat and provides energy (23). Obesity changes the immune cell landscape in adipose tissues, causing the pro-inflammatory cells and CD8+T cells to gradually dominate (24, 25). This phenomenon results in insulin resistance and other related metabolic diseases (26). Obesity also leads to chronic low-grade inflammation and changes in the immune environment of multiple organs (27). Bariatric surgery leads to improvement in obesity-associated comorbidities by decreasing adipose tissue inflammation. The improved inflammatory state following surgery might be explained by the disruption of immuno-inflammatory cascades involving a few crucial molecules which could serve as potential therapeutic targets (28). Obesity is related to cancer risk, with weight loss having a protective function. Cancer causes chronic low-grade inflammation and the disorder of the immune environment (29). A study on obese women undergoing bariatric surgery or a medically supervised low-calorie diet showed how weight loss reduced systemic inflammation and recruitment of protective immune cell types to the endometrium. The study also highlighted how weight loss critically prevented endometrial cancer (30). LSG improves patients’ obesity and related complications by altering the immune microenvironment and energy metabolism pathway.

Competitive endogenous RNA (ceRNA) networks reveal the mechanism of interaction between RNAs and play crucial roles in multiple biological processes. Mounting evidence shows that long non-coding RNAs (lncRNAs) essentially modulate the biological process of diabetic retinopathy (DR). Moreover, it has been demonstrated that ATP2B1-AS1 acts as a miR-4729 sponge to regulate the expression of IQGAP2, reducing high-glucose-induced endothelial dysfunction in DR. This phenomenon has tumor suppressive effects in many cancers, controlling endothelial cells dysfunction (31). Our study identified 20 upregulated, and two downregulated lncRNAs from obese samples before and after receiving LSG treatment; a lncRNA–miRNA–mRNA ceRNA network was constructed with one lncRNA, ten miRNAs, and three biomarkers. In the ceRNA network, both the three signature genes were regulated by lncRNA ATP2B1-AS1. Silencing ATP2B1-AS1 protects mice from myocardial infarction by blocking the NFKBIA mediated NF-κB signaling pathway (19). The IKKβ/NF-κB pathway essentially regulates the inflammatory response and has also recently been implicated in insulin resistance. Alternatively, a reduction in IκBα might cause an increased translocation of NF-κB into the nucleus, increasing the transcription of several inflammatory cytokines, such as tumor necrosis factor-α, associated with insulin resistance. These results confirm that the NF-kappaB/IKKbeta pathway may mediate human obesity-induced insulin resistance. They might serve as diagnostic and prognosis markers as well as therapeutic targets.

Leptin and Adiponectin are two important adipose-regulatory factors in obesity, regulating sugar, fat, and energy metabolism. Leptin inhibits the synthesis of adipose cells, inhibiting appetite and reducing weight. Leptin resistance is a characteristic of human obesity (32). Pathologically, leptin resistance and decreased sensitivity to leptin receptors depolarize islet β cells and promote insulin secretion, resulting in hyperinsulinemia and type 2 diabetes. Moreover, plasma leptin levels are increased in obese type 2 diabetes patients, significantly post-LSG, which indicates the potential of LSG in modifying the conditions of type 2 diabetes by reducing the level of leptin (33). Adiponectin increases insulin sensitivity. Hypoadiponectin was closely related to insulin resistance in obese people; decreasing weight was accompanied by increased adiponectin levels (34), which were negatively correlated with BMI, body fat percentage, waist-to-hip ratio, fasting insulin level, and two hours postprandial blood glucose level, and positively correlated with insulin sensitivity (35).

Our results showed leptin concentration significantly decreased in postoperative patients, while NFKBIA expression level was significantly correlated with leptin. This observation suggested that LSG could reduce leptin levels to improve the postoperative metabolic level of obese patients, leading to increased NFKBIA expression, lowering insulin resistance, and improving insulin sensitivity to improve blood glucose. However, the regulation mechanism of leptin and NFKBIA is still unknown, warranting further systematic and in-depth studies.

Metabolic surgery causes mutual influences and changes in treating obesity (36), reducing the body’s absorption and intake and regulating the level of gastrointestinal hormones, gastrointestinal flora, adipokines, and the central feeding system. Importantly, candidate prognostic biomarkers involved in the ceRNA network were screened out. These biomarkers exhibited essential roles as therapeutic targets and in prognosis analysis in obese patients. Our results also showed that leptin levels decreased significantly after surgery, which may be related to the up-regulation of NFKBIA. However, the regulation mechanism of these two factors needs to be explored in future studies.

## 5 Limitations

There are several limitations of this study. First, the small sample size, further validation in a large number of samples is required. Second, this was based on peripheral blood mononuclear cells which were readily available. Further work on actual adipose tissues is needed to verify expression of characteristic genes. Finally, animal studies would be useful to confirm function of the identified genes.

## Data availability statement

The datasets have been deposited under BioProject ID PRJNA861382.

## Ethics statement

The studies involving human participants were reviewed and approved by Medical Ethics Committee of the First People’s Hospital of Kunming. The patients/participants provided their written informed consent to participate in this study.

## Author contributions

All authors contributed toward data analysis, drafting, and critically revising the paper and agreed to be accountable for all aspects of the work.

## Supplementary material

Supplementary Figure 1 Weighted gene coexpression network analysis (WGCNA) results. **(A)** Clustering of postoperative and preoperative samples. **(B, C)** WGCNA power selection. The horizontal axis represents the soft threshold, and the vertical axis represents the mean value of all gene adjacency functions in the corresponding gene module. **(D)** Modules are segmented by a dynamic tree-cutting algorithm, and a module clustering graph is constructed. **(E)** Set MEDissThres to 0.15 to incorporate similar modules analyzed by the dynamic clipping tree algorithm.

Supplementary Figure 2 Interplay network of three biomarkers through Ingenuity Pathway Analysis (IPA). **(A)** Interplay network of IRF1. **(B)** Interplay network of MFKBIA. **(C)** Interplay network of YRDC.

Supplementary Table 1 91 essential hub genes in the saddle brown modules.

Supplementary Table 2 165 DE-mRNAs between obese patients before and after LSG treatment.

Supplementary Table 3 Functional enrichment analysis of 165 DE-mRNAs.

Supplementary Table 4 Eighteen common genes obtained by overlapping hub genes and DE-mRNAs.

Supplementary Table 5 The enriched terms from the Gene Ontology (GO) analysis.

Supplementary Table 6 The enriched pathways from the Kyoto Encyclopedia of Genes and Genomes pathway (KEGG) analysis.

Supplementary Table 7 The enriched KEGG pathways related to IRF1 from the single-gene Gene set enrichment analysis (GSEA).

Supplementary Table 8 The enriched KEGG pathways related to NFKBIA from the single-gene Gene set enrichment analysis (GSEA).

Supplementary Table 9 The enriched KEGG pathways related to YEDC from the single-gene Gene set enrichment analysis (GSEA).

Supplementary Table 10 22 DE-lncRNAs between obese patients before and after LSG treatment.

Supplementary Table 11 The ceRNA regulatory network of three biomarkers.
