# Supplementary figures and images for: Investigating the change in gene expression profile of blood mononuclear cells post-laparoscopic sleeve gastrectomy in Chinese obese patients
Source: Front Endocrinol (Lausanne). 2023 Mar 14;14:1049484. doi: 10.3389/fendo.2023.1049484 (PMC10043499; doi:10.3389/fendo.2023.1049484)

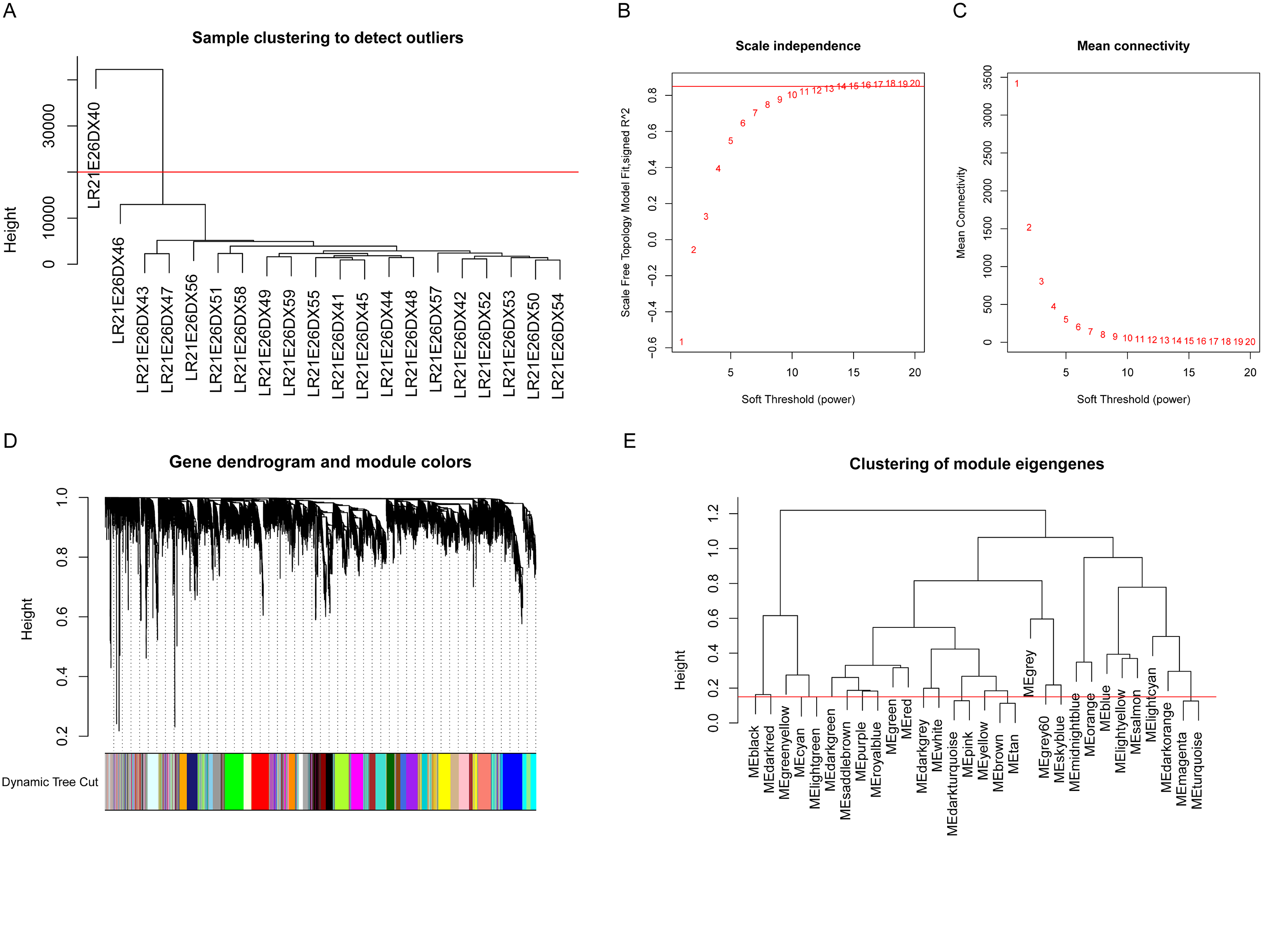

Supplement: Supplementary Figure 1 — Weighted gene coexpression network analysis (WGCNA) results. (A) Clustering of postoperative and preoperative samples. (B, C) WGCNA power selection. The horizontal axis represents the soft threshold, and the vertical axis represents the mean value of all gene adjacency functions in the corresponding gene module. (D) Modules are segmented by a dynamic tree-cutting algorithm, and a module clustering graph is constructed. (E) Set MEDissThres to 0.15 to incorporate similar modules analyzed by the dynamic clipping tree algorithm. [file Image_1.tif]
